# New Coumarin Derivatives and Other Constituents from the Stem Bark of *Zanthoxylum avicennae*: Effects on Neutrophil Pro-Inflammatory Responses

**DOI:** 10.3390/ijms16059719

**Published:** 2015-04-29

**Authors:** Jih-Jung Chen, Chieh-Kai Yang, Yueh-Hsiung Kuo, Tsong-Long Hwang, Wen-Lung Kuo, Yun-Ping Lim, Ping-Jyun Sung, Tsung-Hsien Chang, Ming-Jen Cheng

**Affiliations:** 1 Department of Pharmacy & Graduate Institute of Pharmaceutical Technology, Tajen University, Pingtung 907, Taiwan; E-Mail: jacky.505@yahoo.com.tw; 2 Department of Chinese Pharmaceutical Sciences and Chinese Medicine Resources, China Medical University, Taichung 404, Taiwan; E-Mail: kuoyh@mail.cmu.edu.tw; 3 Department of Biotechnology, Asia University, Taichung 413, Taiwan; 4 Graduate Institute of Natural Products, School of Traditional Chinese Medicine, College of Medicine, Chang Gung University, Taoyuan 333, Taiwan; E-Mail: htl@mail.cgu.edu.tw; 5 Research Center for Industry of Human Ecology, Chang Gung University of Science and Technology, Immunology Consortium, Chang Gung Memorial Hospital, Taoyuan 333, Taiwan; 6 Chung-Jen Junior College of Nursing, Health Sciences and Management, Chiayi 600, Taiwan; E-Mail: m049@cjc.edu.tw; 7 School of Pharmacy, College of Pharmacy, China Medical University, Taichung 404, Taiwan; E-Mail: limyp@mail2000.com.tw; 8 National Museum of Marine Biology and Aquarium, Pingtung 944, Taiwan; E-Mail: pjsung@nmmba.gov.tw; 9 Department of Medical Education and Research, Kaohsiung Veterans General Hospital, Kaohsiung 813, Taiwan; E-Mail: hangth@vghks.gov.tw; 10 Bioresource Collection and Research Center (BCRC), Food Industry Research and Development Institute (FIRDI), Hsinchu 300, Taiwan; E-Mail: cmj@firdi.org.tw

**Keywords:** *Zanthoxylum avicennae*, Rutaceae, coumarin, structure elucidation, anti-inflammatory activity

## Abstract

Three new coumarin derivatives, 8-formylalloxanthoxyletin (**1**), avicennone (**2**), and (*Z*)-avicennone (**3**), have been isolated from the stem bark of *Zanthoxylum avicennae* (*Z. avicennae*), together with 15 known compounds (**4**–**18**). The structures of these new compounds were determined through spectroscopic and MS analyses. Compounds **1**, **4**, **9**, **12**, and **15** exhibited inhibition (half maximal inhibitory concentration (IC_50_) values ≤7.65 µg/mL) of superoxide anion generation by human neutrophils in response to formyl-l-methionyl-l-leucyl-l-phenylalanine/cytochalasin B (fMLP/CB). Compounds **1**, **2**, **4**, **8** and **9** inhibited fMLP/CB-induced elastase release with IC_50_ values ≤8.17 µg/mL. This investigation reveals bioactive isolates (especially **1**, **2**, **4**, **8**, **9**, **12** and **15**) could be further developed as potential candidates for the treatment or prevention of various inflammatory diseases.

## 1. Introduction

*Zanthoxylum avicennae* (Lam.) DC. (Rutaceae) is an evergreen shrub distributed in Vietnam, Philippines, southern China, and Taiwan [1]. Neolignans [2], alkaloids [2,3,4], coumarins [3,4], flavonoids [3], benzenoids [3,4], and their derivatives were isolated from this plant in previous studies. Many of these compounds were found to exhibit anti-inflammatory [2,3], anti-platelet aggregation [5], and anti-hepatitis B virus (HBV) [6] activities.

**Figure 1:**
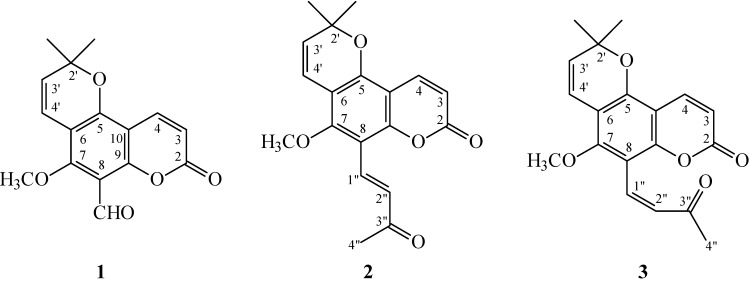
The chemical structures of new compounds **1**–**3** isolated from *Z. avicennae*.

**Figure 2:**
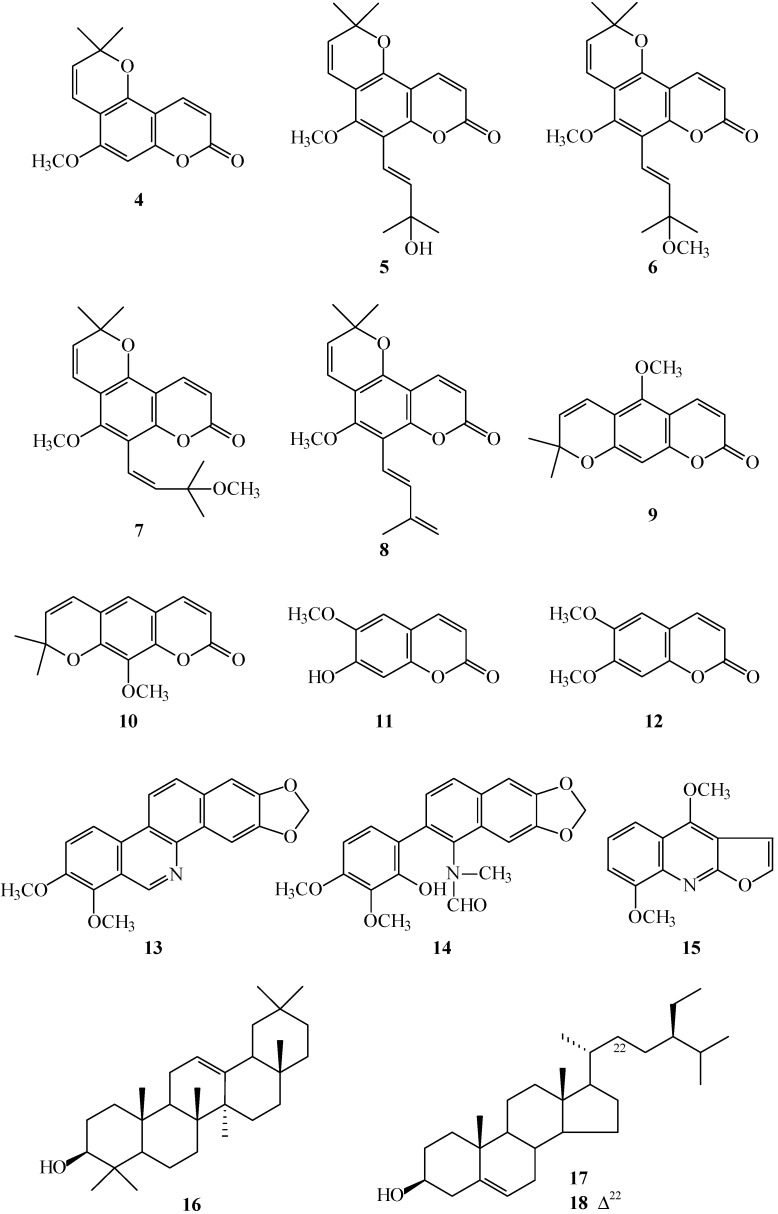
The chemical structures of known compounds **4**–**18** isolated from *Z. avicennae*.

In our studies on the anti-inflammatory constituents of Formosan plants, many species have been screened for *in vitro* inhibitory activity on neutrophil pro-inflammatory responses, and *Z. avicennae* has been found to be an active species. The MeOH extract of stem bark of *Z. avicennae* showed potent inhibitory effects on superoxide anion generation and elastase release by human neutrophils in response to formyl-l-methionyl-l-leucyl-l-phenylalanine/cytochalasin B (fMLP/CB). Figure 1 illustrates the structures of three new coumarins, 8-formylalloxanthoxyletin (**1**), avicennone (**2**), and (*Z*)-avicennone (**3**). Fifteen known compounds (**4**–**18**), have been isolated and identified from the stem bark of *Z. avicennae* and their structures are depicted in Figure 2.

This paper describes the structural elucidation of the compounds numbered **1** through **3**, and the inhibitory activities of all isolates on superoxide generation and elastase release by neutrophils.

## 2. Results

Chromatographic purification of the EtOAc-soluble fraction of a MeOH extract of stem bark of *Z. avicennae* on a silica gel column and preparative thin-layer chromatography (TLC) afforded three new (**1**–**3**) and fifteen known compounds (**4**–**18**).

**Figure 3:**
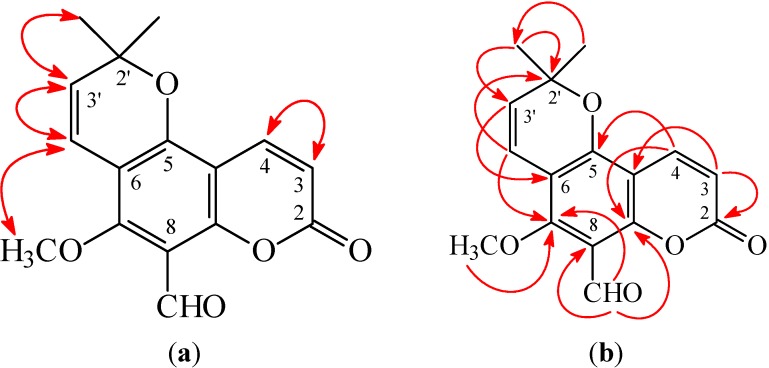
Key nuclear Overhauser effect spectrometry (NOESY) (**a**) and heteronuclear multiple-bond correlation (HMBC) (**b**) correlations of **1**.

**Figure 4:**
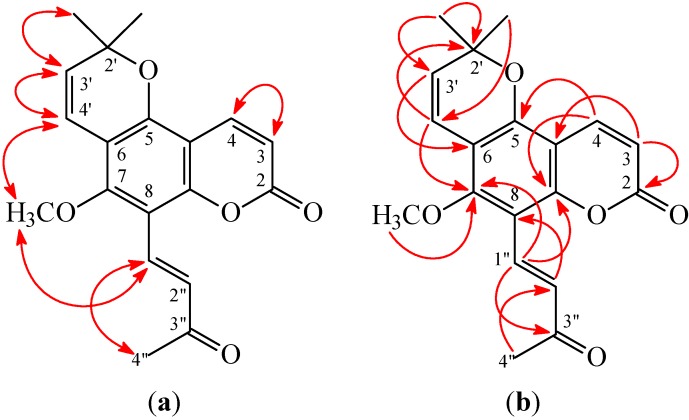
Key NOESY (**a**) and HMBC (**b**) correlations of **2**.

**Figure 5:**
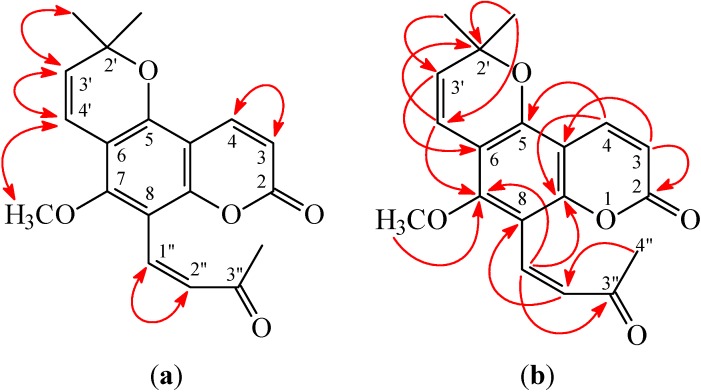
Key NOESY (**a**) and HMBC (**b**) correlations of **3**.

8-Formylalloxanthoxyletin (**1**) was isolated as amorphous powder with molecular formula C_16_H_14_O_5_ as determined by positive-ion high-resolution electrospray ionization (HR-ESI-MS), showing an [M + Na]^+^ ion at *m*/*z* 309.0739 (calculated for C_16_H_14_O_5_Na, 309.0739). The presence of carbonyl groups was revealed by the bands at 1693, 1729 cm^−1^ in the Infrared (IR) spectrum, and was confirmed by the resonances at δ 159.4, 185.7 in the ^13^C NMR spectrum. The ^1^H NMR spectrum of **1** showed the presence of a 2,2-dimethyl-2*H*-pyran moiety [δ 1.53 (6H, s, Me-2' × 2), 5.70, 6.64 (each 1H, each d, *J* = 10.5 Hz, H-3' and H-4')], a methoxy group [δ 3.92 (3H, s, OMe-7)], a formyl group [δ 10.53 (1H, s, CHO-8)], and the typical H*-*3 and H*-*4 protons of the coumarin nucleus [δ 6.32, 8.00 (each 1H, each d, *J* = 10.0 Hz, H*-*3 and H-4)]. The ^1^H NMR spectrum of **1** was similar to those of alloxanthoxyletin (**4**) [7], except that the 8-formyl group [δ 10.53 (1H, s)] of **1** replaced H-8 of alloxanthoxyletin (**4**). This was supported by HMBC correlation observed between CHO-8 (δ 10.53) and C-7 (δ 160.2), C-8 (δ 110.7), and C-9 (δ 157.5). The full assignment of ^1^H and ^13^C NMR resonances was confirmed by ^1^H–^1^H correlation spectroscopy (COSY), nuclear Overhauser effect spectrometry (NOESY) (Figure 3), Distortionless Enhancement by Polazization Transfer (DEPT), eteronuclear single-quantum coherence (HSQC), and heteronuclear multiple-bond correlation (HMBC) (Figure 3) techniques. According to the evidence above, the structure of **1** was elucidated as 5-methoxy-2,2-dimethyl-8-oxo-2,8-dihydropyrano [2,3-*f*]chromene-6-carbaldehyde, named 8-formylalloxanthoxyletin.

(*E*)-Avicennone (**2**) was obtained as amorphous powder. Its molecular formula, C_19_H_18_O_5_, was determined on the basis of the positive HRESIMS at *m*/*z* 349.1056 [M + Na]^+^ (calcd 349.1052) and supported by the ^1^H, ^13^C, and DEPT NMR data. The presence of carbonyl groups was revealed by the bands at 1695 and 1734 cm^−1^ in the IR spectrum, and was confirmed by the resonances at δ 160.0 and 199.8 in the ^13^C NMR spectrum. The ^1^H NMR spectrum of **2** displayed the presence of a 2,2-dimethyl-2*H*-pyran moiety [δ 1.51 (6H, s, Me-2' × 2), 5.70, 6.59 (each 1H, each d, *J* = 10.2 Hz, H-3' and H-4')], a methoxy group [δ 3.81 (3H, s, OMe-7)], an (*E*)-3-oxobut-1-enyl group [δ 2.42 (3H, s, Me-3''), 7.32, 7.83 (each 1H, each d, *J* = 16.5 Hz, H-2'' and H-1'')], and the typical H*-*3 and H*-*4 protons of the coumarin nucleus [δ 6.30, 8.02 (each 1H, each d, *J* = 9.6 Hz, H*-*3 and H-4)]. The ^1^H NMR data of **2** were similar to 8-formylalloxanthoxyletin (**1**), except that the (*E*)-3-oxobut-1-enyl group at C-8 of **2** replaced 8-formyl group of 8-formylalloxanthoxyletin (**1**). This was supported by the NOESY correlations between H-1'' (δ 7.83) and H-4'' (δ 2.42) and OMe-7 (δ 3.81) and by the HMBC correlation between H-1'' (δ 7.83) and C-7 (δ 159.2), C-8 (δ 107.5), C-9 (δ 154.1), and C-3'' (δ 199.8) of **2**. In addition, the (*E*)-configuration of **2** was established by the following evidences: (a) The larger coupling constant (*J* = 16.5 Hz) between H-1'' and H-2'' of **2**; (b) The NOESY correlations were observed between H-1'' (δ 7.83) and H-4'' (δ 2.42) (Figure 3). Thus, (*E*)-avicennone was elucidated as structure **2**. This structure was confirmed by the ^1^H-^1^H COSY, NOESY (Figure 4), DEPT, HSQC, and HMBC techniques (Figure 4).

(*Z*)-Avicennone (**3**) was isolated as amorphous powder. The molecular formula C_19_H_18_O_5_ was deduced from a sodium adduct ion at *m*/*z* 349.1054 [M + Na]^+^ (calcd 349.1052) in the HR-ESI mass spectrum. The presence of carbonyl groups was revealed by the bands at 1694, 1734 cm^−1^ in the IR spectrum. The ^1^H NMR spectrum of **3** illustrated the presence of a 2,2-dimethyl-2*H*-pyran moiety [δ 1.49 (6H, s, Me-2' × 2), 5.64, 6.56 (each 1H, each d, *J* = 10.2 Hz, H-3' and H-4')], a methoxy group [δ 3.72 (3H, s, OMe-7)], an (*Z*)-3-oxobut-1-enyl group [δ 2.23 (3H, s, Me-3''), 6.40, 6.79 (each 1H, each d, *J* = 12.6 Hz, H-2'' and H-1'')], and the typical H*-*3 and H*-*4 protons of the coumarin nucleus [δ 6.23, 7.99 (each 1H, each d, *J* = 9.6 Hz, H*-*3 and H-4)]. The ^1^H and ^13^C NMR data of **3** were similar to those of (*E*)-avicennone (**2**), except that the (*Z*)-3-oxobut-1-enyl group [δ_H_ 2.23 (3H, s, Me-3''), 6.40, 6.79 (each 1H, each d, *J* = 12.6 Hz, H-2'' and H-1''); δ_C_ 29.7 (C-4''), 128.6 (C-1''), 132.3 (C-2''), 200.6 (C-3'')] at C-8 of **3** replaced the (*E*)-3-oxobut-1-enyl group at C-8 of (*E*)-avicennone (**2**). This was supported by the coupling constant (*J* = 12.6 Hz) between H-1'' and H-2'' of **3** and by the NOESY correlations between H-1'' (δ 6.79) and H-2'' (δ 6.40). According to the above data, the structure of **3** was elucidated as (*Z*)-avicennone. This was supported by ^1^H–^1^H COSY and NOESY (Figure 5) experiments, and ^13^C NMR assignments were confirmed by DEPT, HSQC, and HMBC (Figure 5) techniques.

The known isolates were readily identified by a comparison of physical and spectroscopic data (UV, IR, ^1^H NMR, [α]_D_, and MS) with corresponding authentic samples or literature values, and this included nine coumarins, alloxanthoxyletin (**4**) [7], avicennol (**5**) [8], avicennol methyl ether (**6**) [9], *cis*-avicennol methyl ether (**7**) [9], avicennin (**8**) [8] [9], xanthoxyletin (**9**) [7], luvangetin (**10**) [10], scopoletin (**11**) [11], and aesculetin dimethyl ether (**12**) [12]; two benzo[*c*]phenanthridine derivatives, norchelerythrine (**13**) [13] and arnottianamide (**14**) [14]; a furoquinoline, γ-fagarine (**15**) [14]; a triterpene, β-amyrin (**16**) [15]; and two steroids, β-sitosterol (**17**) [16] and stigmasterol (**18**) [16].

Granule proteases (e.g., elastase, cathepsin G, and proteinase-3) and reactive oxygen species (ROS) (e.g., superoxide anion (O_2_^•−^) and hydrogen peroxide) produced by human neutrophils are involved in the pathogenesis of a variety of inflammatory diseases. The effects on neutrophil pro-inflammatory responses of compounds isolated from the stem bark of *Z. avicennae* were evaluated by suppressing fMet-Leu-Phe/cytochalasin B (fMLP/CB)-induced superoxide anion (O_2_^•−^) generation and elastase release by human neutrophils. The inhibitory activity data on neutrophil pro-inflammatory responses are summarized in Table 1. Diphenyleneiodonium and phenylmethylsulfonyl fluoride were used as positive controls for O_2_^•−^ generation and elastase release, respectively. From the results of our biological tests, the following conclusions can be drawn: (a) 8-Formylalloxanthoxyletin (**1**), alloxanthoxyletin (**4**), xanthoxyletin (**9**), aesculetin dimethyl ether (**12**), and γ-fagarine (**15**) exhibited potent inhibition (IC_50_ ≤ 7.65 µg/mL) of superoxide anion (O_2_^•−^) generation by human neutrophils in response to fMLP/CB; (b) 8-Formylalloxanthoxyletin (**1**), (*E*)-avicennone (**2**), alloxanthoxyletin (**4**), avicennin (**8**), and xanthoxyletin (**9**) exhibited potent inhibition (IC_50_ ≤ 8.17 µg/mL) against fMLP-induced elastase release; (c) Among the coumarins with a 2,2-dimethyl-2*H*-pyran moiety at C-6,7, xanthoxyletin (**9**) (with a 5-methoxy group) exhibited more effective inhibition than its analogue, luvangetin (**10**) (with a 8-methoxy group) against fMLP-induced O_2_^•−^ generation and elastase release; (d) Among the coumarins (**1**–**8**) with a 7-methoxy group and a 2,2-dimethyl-2*H*-pyran moiety at C-5,6, 8-formylalloxanthoxyletin (**1**) (with a 8-formyl group) and alloxanthoxyletin (**4**) (without any substituted group at C-8) exhibited more effective inhibition than its analogues (**2**, **3**, and **5**–**8**) against fMLP-induced O_2_^•−^ generation and elastase release; (e) Alloxanthoxyletin (**4**) and xanthoxyletin (**9**) were the most effective among these compounds, with IC_50_ values of 1.47 ± 0.53 and 1.47 ± 0.41 µg/mL, respectively, against fMLP-induced superoxide anion generation; and (f) 8-Formylalloxanthoxyletin (**1**) exhibited the most effective among the isolates, with IC_50_ value of 2.59 ± 0.52 µg/mL, against fMLP-induced elastase release.

**Table 1:** Inhibitory effects of compounds **1**–**18** from the stem bark of *Z. avicennae* on superoxide radical anion generation and elastase release by human neutrophils in response to fMet-Leu-Phe/cytochalasin B ^a^.

Compounds	Superoxide Anion	Elastase
	IC_50_ [µg/mL] ^b^ or (Inh %) ^c^	
8-Formylalloxanthoxyletin (**1**)	4.60 ± 0.83 ^d^	2.59 ± 0.52 ^d^
(*E*)-Avicennone (**2**)	(24.4 ± 5.1) ^e^	8.17 ± 1.61 ^d^
(*Z*)-Avicennone (**3**)	(18.0 ± 3.1)	(40.2 ± 4.5) ^f^
Alloxanthoxyletin (**4**)	1.47 ± 0.53 ^d^	3.43 ± 0.63 ^d^
Avicennol (**5**)	(27.6 ± 4.8) ^e^	10.9 ± 1.4 ^d^
Avicennol methyl ether (**6**)	(28.6 ± 1.8) ^d^	(6.31 ± 3.50)
*cis*-Avicennol methyl ether (**7**)	(23.2 ± 2.5) ^e^	(6.51 ± 1.22) ^f^
Avicennin (**8**)	(38.5 ± 4.2) ^d^	6.21 ± 1.60 ^d^
Xanthoxyletin (**9**)	1.47 ± 0.41 ^d^	4.18 ± 0.73 ^d^
Luvangetin (**10**)	(29.0 ± 3.7) ^e^	(5.28 ± 1.30) ^f^
Aesculetin dimethyl ether (**11**)	7.65 ± 1.62	(45.8 ± 5.1)
Scopoletin (**12**)	(25.6 ± 4.8)	(43.7 ± 3.2)
Norchelerythrine (**13**)	(12.1 ± 2.2) ^e^	(23.8 ± 4.5) ^e^
Arnottianamide (**14**)	(22.8 ± 4.2)	(16.5 ± 5.1)
γ-Fagarine (**15**)	6.85 ± 0.46 ^f^	(21.3 ± 3.5)
β-Amyrin (**16**)	(8.75 ± 4.14)	(2.62 ± 1.61)
Mixture of β-Sitosterol (**17**) and Stigmasterol (**18**)	(1.25 ± 1.03)	(2.24 ± 1.14)
Diphenyleneiodonium ^g^	0.53 ± 0.22 ^d^	–
Phenylmethylsulfonyl fluoride ^g^	–	34.2 ± 5.6 ^d^

^a^ Results are presented as averages ± SEM (*n* = 4); ^b^ Concentration necessary for 50% inhibition (IC_50_). If IC_50_ value of compound was <10 µg/mL, it was displayed as IC_50_ [µg/mL]; ^c^ Percentage of inhibition (Inh %) at 10 µg/mL. If IC_50_ value of compound was ≥10 µg/mL, it was shown as (Inh %) at 10 µg/mL; ^d^ *p* < 0.001 compared with the control; ^e^ *p* < 0.01 compared with the control; ^f^ *p* < 0.05 compared with the control; ^g^ Diphenyleneiodonium and phenylmethylsulfonyl were used as positive controls for superoxide anion generation and elastase release, respectively.

## 3. Discussion

Eighteen compounds, including three new coumarins **1**–**3**, were isolated from the stem bark of *Z. avicennae*. Known compounds **4**, **7**, **9**, **14**, **16**, and **18** were obtained from this plant for the first time. The structures of these compounds were established on the basis of spectroscopic data. Discovery of more new coumarins from the genus *Zanthoxylum* may not only provide more structure-activity data of coumarins, but may also contribute to enhancing our understanding of the taxonomy and evolution of the genus *Zanthoxylum*.

Granule proteases (e.g., elastase, cathepsin G) and reactive oxygen species (ROS) (e.g., superoxide anion (O_2_^•−^), hydrogen peroxide) and produced by human neutrophils contribute to the pathogenesis of inflammatory diseases. Inhibition of the inappropriate activation of neutrophils by drugs has been proposed as a way to ameliorate inflammatory diseases. Based on the results of our biological tests (Table 1), xanthoxyletin (**9**), alloxanthoxyletin (**4**), and 8-formylalloxanthoxyletin (**1**) were the most effective among these compounds, with IC_50_ values of 1.47 ± 0.41, 1.47 ± 0.53, and 4.60 ± 0.83 µg/mL, respectively, against fMLP-induced superoxide anion generation. 8-Formylalloxanthoxyletin (**1**), alloxanthoxyletin (**4**), and xanthoxyletin (**9**) exhibited the most effective among the isolates, with IC_50_ values of 2.59 ± 0.52, 3.43 ± 0.63, and 4.18 ± 0.73 µg/mL, respectively, against fMLP-induced elastase release. Our study suggests *Z. avicennae* and its isolates (especially **1**, **4**, and **9**) could be further developed as potential candidates for the treatment or prevention of various inflammatory diseases. More experiments should be performed to deduce the action modes of these compounds.

## 4. Experimental Section

### 4.1. Ethics Statement

Blood was taken from healthy human donors (20–30 years old) by venipuncture, using a protocol (No. 102-1595A3) approved by the Institutional Review Board at Chang Gung Memorial Hospital. All donors gave written consent. The Medical Ethics Committee of Chang Gung Memorial Hospital approved this consent procedure.

### 4.2. General Experimental Procedures

Melting points were determined on a Yanaco micro-melting point apparatus and were uncorrected. Optical rotations were measured using a Jasco DIP-370 polarimeter (Japan Spectroscopic Corporation, Tokyo, Japan) in CHCl_3_. Ultraviolet (UV) spectra were obtained on a Jasco UV-240 spectrophotometer (Japan Spectroscopic Corporation). Infrared (IR) spectra (neat or KBr) were recorded on a Perkin Elmer 2000 FT-IR spectrometer (Perkin Elmer Corporation, Norwalk, CT, USA). Nuclear magnetic resonance (NMR) spectra, including correlation spectroscopy (COSY), nuclear Overhauser effect spectrometry (NOESY), heteronuclear multiple-bond correlation (HMBC), and heteronuclear single-quantum coherence (HSQC) experiments, were acquired using Varian INOVA-500 or Varian VNMRS-600 spectrometer (Varian Inc., Palo Alto, CA, USA) operating at 500 or 600 MHz (^1^H) and 125 or 150 MHz (^13^C), respectively, with chemical shifts given in ppm (δ) using tetramethylsilane (TMS) as an internal standard. Electrospray ionisation (ESI) and high-resolution electrospray ionization (HRESI)-mass spectra were recorded on a Bruker APEX II (Bruker, Bremen, Germany) or a VG Platform Electrospray ESI/MS mass spectrometer (Fison, Villeurbanne, France). Silica gel (70–230, 230–400 mesh, Merck, Darmstadt, Germany) was used for column chromatography (CC). Silica gel 60 F-254 (Merck, Darmstadt, Germany) was used for thin-layer chromatography (TLC) and preparative thin-layer chromatography (PTLC).

### 4.3. Plant Material

The stem bark of *Z. avicennae* was collected from Yanpu, Pingtung County, Taiwan, in June 2006 and identified by Jih-Jung Chen. A voucher specimen (ZA-200606) was deposited in the Department of Pharmacy, Tajen University, Pingtung, Taiwan.

### 4.4. Extraction and Isolation

The dried stem bark (8.75 kg) of *Z. avicennae* was pulverized and extracted three times with MeOH (40 L each) for 3 days. The MeOH extracts were concentrated under reduced pressure at 35 °C, and the residue (550 g) was partitioned between EtOAc and H_2_O (1:1). The EtOAc layer was concentrated to give a residue (fraction A, 302.8 g). The water layer was further extracted with *n*-BuOH, and the *n*-BuOH-soluble part (fraction B, 91.1 g) and the water-solubles (fraction C, 127.3 g) were separated. Fraction A (115 g) was chromatographed on silica gel (70–230 mesh, 5.2 kg), eluting with CH_2_Cl_2_, gradually increasing the polarity with MeOH to give 14 fractions: A1 (10 L, CH_2_Cl_2_), A2 (4 L, CH_2_Cl_2_/MeOH, 99:1), A3 (5 L, CH_2_Cl_2_/MeOH, 95:1), A4 (5 L, CH_2_Cl_2_/MeOH, 90:1), A5 (2 L, CH_2_Cl_2_/MeOH, 80:1), A6 (3 L, CH_2_Cl_2_/MeOH, 70:1), A7 (4 L, CH_2_Cl_2_/MeOH, 50:1), A8 (7.5 L, CH_2_Cl_2_/MeOH, 30:1), A9 (2 L, CH_2_Cl_2_/MeOH, 20:1), A10 (4 L, CH_2_Cl_2_/MeOH, 10:1), A11 (4 L, CH_2_Cl_2_/MeOH, 5:1), A12 (3 L, CH_2_Cl_2_/MeOH, 3:1), A13 (2 L, CH_2_Cl_2_/MeOH, 1:1), A14 (2 L, MeOH). Fraction A2 (11.8 g) was chromatographed further on silica gel (230–400 mesh, 535 g) eluting with *n*-hexane/EtOAc (3:1–0:1) to give 14 fractions (each 500 mL, A2-1–A2-14). Part (112 mg) of fraction A2-13 was purified by preparative TLC (silica gel, CH_2_Cl_2_/acetone, 30:1) to afford **12** (3.8 mg) (R*_f_* = 0.70). Part (96 mg) of fraction A2-14 was purified by preparative TLC (silica gel, CH_2_Cl_2_/acetone, 25:1) to obtain **15** (3.3 mg) (R*_f_* = 0.60). Fraction A4 (9.1 g) was chromatographed further on silica gel (230–400 mesh, 425 g) eluting with *n*-hexane/EtOAc (3:1–0:1) to give 13 fractions (each 1 L, A4-1–A4-13). Fraction A4-1 (273 mg) was purified by CC (silica gel, *n*-hexane/acetone, 2:1–0:1) to afford 11 subfractions (each 200 mL, A4-1-1–A4-1-11). Fraction A4-1-9 (33 mg) was purified by preparative TLC (silica gel, CHCl_3_/EtOAc, 5:1) to give **11** (3.8 mg) (R*_f_* = 0.50). Part (140 mg) of fraction A4-7 was purified by preparative TLC (silica gel, CHCl_3_/acetone, 40:1) to obtain **7** (3.7 mg) (R*_f_* = 0.47). Part (148 mg) of fraction A4-9 was purified by preparative TLC (silica gel, *n*-hexane/EtOAc, 5:1) to yield **1** (3.5 mg) (R*_f_* = 0.24). Fraction A5 (8.8 g) was chromatographed further on silica gel (230–400 mesh, 405 g) eluting with *n*-hexane/EtOAc (10:1–0:1) to give 21 fractions (each 1 L, A5-1–A5-21). Part (81 mg) of fraction A5-8 was purified by preparative TLC (silica gel, *n*-hexane/EtOAc, 3:1) to afford **16** (6.0 mg) (R*_f_* = 0.71). Part (72 mg) of fraction A5-11 was purified by preparative TLC (silica gel, *n*-hexane/CHCl_3_, 1:3) to yield **9** (20.1 mg) (R*_f_* = 0.33). Fraction A5-14 (225 mg) was purified by CC (silica gel, CH_2_Cl_2_/acetone, 45:1) to afford 5 subfractions (each 200 mL, A5-14-1–A5-14-5). Fraction A5-14-2 (28 mg) was purified by preparative TLC (silica gel, *n*-hexane/EtOAc, 5:1) to give **4** (10.2 mg) (R*_f_* = 0.35). Fraction A5-14-3 (67 mg) was purified by preparative TLC (silica gel, *n*-hexane/EtOAc, 5:1) to obtain **6** (40.1 mg) (R*_f_* = 0.30). Part (118 mg) of fraction A5-17 was purified by preparative TLC (silica gel, *n*-hexane/EtOAc, 2:1) to afford **10** (77 mg) (R*_f_* = 0.40). Fraction A6 (10.5 g) was chromatographed further on silica gel (230–400 mesh, 478 g) eluting with *n*-hexane/acetone (2:1–0:1) to give 11 fractions (each 500 mL, A6-1–A6-11). Part (125 mg) of fraction A6-1 was purified by preparative TLC (silica gel, CHCl_3_/acetone, 80:1) to obtain **2** (3.9 mg) (R*_f_* = 0.87) and **3** (2.8 mg) (R*_f_* = 0.88). Fraction A7 (9.2 g) was chromatographed further on silica gel (230–400 mesh, 420 g) eluting with *n*-hexane/EtOAc (6:1–0:1) to give 17 fractions (each 500 mL, A7-1–A7-17). Part (136 mg) of fraction A7-7 was purified by preparative TLC (silica gel, *n*-hexane/EtOAc, 4:1) to obtain mixture of **17** and **18** (14.1 mg) (R*_f_* = 0.50). Part (135 mg) of fraction A7-8 was purified by preparative TLC (silica gel, *n*-hexane/EtOAc, 3:1) to yield **8** (10.2 mg) (R*_f_* = 0.46). Part (125 mg) of fraction A7-9 was purified by preparative TLC (silica gel, CHCl_3_/EtOAc, 15:1) to give **5** (3.5 mg) (R*_f_* = 0.79). Part (95 mg) of fraction A7-13 was purified by preparative TLC (silica gel, *n*-hexane/EtOAc, 2:1) to yield **13** (4.3 mg) (R*_f_* = 0.48). Part (118 mg) of fraction A7-15 was purified by preparative TLC (silica gel, *n*-hexane/EtOAc, 2:3) to give **14** (5.3 mg) (R*_f_* = 0.62).

#### 4.4.1. 8-Formylalloxanthoxyletin (**1**)

Amorphous powder. UV (MeOH): λ_max_ (log ε) = 224 (4.39), 261 (4.15), 281 (4.11) nm. IR (KBr): υ_max_ = 1729 (C=O), 1693 (C=O) cm^−1^. ^1^H NMR (CDCl_3_, 500 MHz): δ = 1.53 (6H, s, Me-2' × 2), 3.92 (3H, s, OMe-7), 5.70 (1H, d, *J* = 10.5 Hz, H-3'), 6.32 (1H, d, *J* = 10.0 Hz, H-3), 6.64 (1H, d, *J* = 10.5 Hz, H-4'), 8.00 (1H, d, *J* = 10.0 Hz, H-4), 10.53 (1H, s, CHO) (Figure S1). ^13^C NMR (CDCl_3_, 125 MHz): δ = 28.5 (Me-2' × 2), 63.6 (OMe), 79.7 (C-2'), 105.5 (C-10), 110.7 (C-6), 110.7 (C-8), 113.5 (C-3), 115.4 (C-4'), 129.3 (C-3'), 137.8 (C-4), 155.3 (C-5), 157.5 (C-9), 159.4 (C-2), 160.2 (C-7), 185.7 (CHO) (Figure S2). ESI-MS: *m*/*z* = 309 [M + Na]^+^ (Figure S3). HR-ESI-MS: *m*/*z* = 309.0739 [M + Na]^+^ (calcd for C_16_H_14_O_5_Na: 309.0739) (Figure S4).

#### 4.4.2. (*E*)-Avicennone (**2**)

Amorphous powder. UV (MeOH): λ_max_ (log ε) = 227 (4.00), 281 (4.16) nm. IR (neat): υ_max_ = 1734 (C=O), 1695 (C=O) cm^−1^. ^1^H NMR (CDCl_3_, 600 MHz): δ = 1.51 (6H, s, Me-2' × 2), 2.42 (3H, s, Me-3''), 3.81 (3H, s, OMe-7), 5.70 (1H, d, *J* = 10.2 Hz, H-3'), 6.30 (1H, d, *J* = 9.6 Hz, H-3), 6.59 (1H, d, *J* = 10.2 Hz, H-4'), 7.32 (1H, d, *J* = 16.5 Hz, H-2''), 7.83 (1H, d, *J* = 16.5 Hz, H-1''), 8.02 (1H, d, *J* = 9.6 Hz, H-4) (Figure S5). ^13^C NMR (CDCl_3_, 150 MHz): δ = 28.2 (Me-2' × 2), 29.7 (C-4''), 62.4 (OMe), 78.7 (C-2'), 106.3 (C-10), 107.5 (C-8), 110.8 (C-6), 113.2 (C-3), 116.1 (C-4'), 129.5 (C-3'), 130.3 (C-2''), 131.6 (C-1''), 138.2 (C-4), 149.7 (C-5), 154.1 (C-9), 159.2 (C-7), 160.0 (C-2), 199.8 (C-3'') (Figure S6). ESI-MS: *m*/*z* = 349 [M + Na]^+^ (Figure S7). HR-ESI-MS: *m*/*z* = 349.1056 [M + Na]^+^ (calcd for C_19_H_18_O_5_Na, 349.1052) (Figure S8).

#### 4.4.3. (*Z*)-Avicennone (**3**)

Amorphous powder. UV (MeOH): λ_max_ (log ε) = 226 (4.01), 280 (4.14) nm. IR (KBr): υ_max_ = 1734 (C=O), 1694 (C=O) cm^−1^. ^1^H NMR (CDCl_3_, 600 MHz): δ = 1.49 (6H, s, Me-2' × 2), 2.23 (3H, s, Me-3''), 3.72 (3H, s, OMe-7), 5.64 (1H, d, *J* = 10.2 Hz, H-3'), 6.23 (1H, d, *J* = 9.6 Hz, H-3), 6.40 (1H, d, *J* = 12.6 Hz, H-2''), 6.56 (1H, d, *J* = 10.2 Hz, H-4'), 6.79 (1H, d, *J* = 12.6 Hz, H-1''), 7.99 (1H, d, *J* = 9.6 Hz, H-4) (Figure S9). ^13^C NMR (CDCl_3_, 150 MHz): δ = 28.2 (Me-2' × 2), 29.7 (C-4''), 62.4 (OMe), 77.8 (C-2'), 105.9 (C-10), 107.9 (C-8), 110.3 (C-6), 113.0 (C-3), 116.2 (C-4'), 128.6 (C-1''), 129.1 (C-3'), 132.3 (C-2''), 138.2 (C-4), 148.9 (C-5), 152.6 (C-9), 156.9 (C-7), 159.8 (C-2), 200.6 (C-3'') (Figure S10). ESI-MS: *m*/*z* = 349 [M + Na]^+^ (Figure S11). HR-ESI-MS: *m*/*z* = 349.1054 [M + Na]^+^ (calcd for C_19_H_18_O_5_Na, 349.1052) (Figure S12).

### 4.5. Biological Assay

The effect of the isolated compounds on neutrophil pro-inflammatory response was evaluated by monitoring the inhibition of superoxide anion generation and elastase release in fMLP/CB-activated human neutrophils in a concentration-dependent manner. The purity of the tested compounds was >98% as identified by NMR and MS.

#### 4.5.1. Preparation of Human Neutrophils

Human neutrophils from venous blood of healthy, adult volunteers (20–28 years old) were isolated using a standard method of dextran sedimentation prior to centrifugation in a Ficoll Hypaque gradient and hypotonic lysis of erythrocytes [17]. Purified neutrophils containing > 98% viable cells, as determined by the trypan blue exclusion method [18], were re-suspended in a calcium (Ca^2+^)-free HBSS buffer at pH 7.4 and were maintained at 4 °C prior to use.

#### 4.5.2. Measurement of Superoxide Anion Generation

The assay for measurement of superoxide anion generation was based on the SOD-inhibitable reduction of ferricytochrome *c* [19,20]. In brief, after supplementation with 0.5 mg/mL ferricytochrome *c* and 1 mM Ca^2+^, neutrophils (6 × 10^5^/mL) were equilibrated at 37 °C for 2 min and incubated with different concentrations (10–0.01 μg/mL) of compounds or DMSO (as control) for 5 min. Cells were incubated with cytochalasin B (1 μg/mL) for 3 min prior to the activation with 100 nM formyl-l-methionyl-l-leucyl-lphenylalanine for 10 min. Changes in absorbance with the reduction of ferricytochrome *c* at 550 nm were continuously monitored in a double-beam, six-cell positioner spectrophotometer with constant stirring (Hitachi U-3010, Tokyo, Japan). Calculations were based on differences in the reactions with and without SOD (100 U/mL) divided by the extinction coefficient for the reduction of ferricytochrome *c* (ε = 21.1/mM/10 mm).

#### 4.5.3. Measurement of Elastase Release

Degranulation of azurophilic granules was determined by measuring elastase release as described previously [19,20]. Experiments were performed using MeO-Suc-Ala-Ala-Pro-Val-*p*-nitroanilide as the elastase substrate. Briefly, after supplementation with MeO-Suc-Ala-Ala-Pro-Val-*p*-nitroanilide (100 μM), neutrophils (6 × 10^5^/mL) were equilibrated at 37 °C for 2 min and incubated with compounds for 5 min. Cells were stimulated with fMLP (100 nM)/CB (0.5 μg/mL), and changes in absorbance at 405 nm were monitored continuously in order to assay elastase release. The results were expressed as the percent of elastase release in the fMLP/CB-activated, drug-free control system.

#### 4.5.4. Statistical Analysis

Results are expressed as the mean ± standard error of the mean (SEM), and comparisons were made using Student’s *t*-test. A probability of 0.05 or less was considered significant. The software SigmaPlot was used for the statistical analysis.

## 5. Conclusions

Eighteen compounds, including three new coumarins (**1**–**3**), were isolated from the stem bark of *Z. avicennae*. The structures of these compounds were established on the basis of spectroscopic data. Reactive oxygen species (ROS) (e.g., superoxide anion (O_2_^•−^), hydrogen peroxide) and granule proteases (e.g., elastase, cathepsin G) produced by human neutrophils contribute to the pathogenesis of inflammatory diseases. The effects on neutrophil pro-inflammatory responses of isolates were evaluated by suppressing fMLP/CB-induced O_2_^•−^ generation and elastase release by human neutrophils. The results of anti-inflammatory experiments indicate that compounds **1**, **2**, **4**, **9**, **11**, and **15** can significantly inhibit fMLP-induced O_2_^•−^ generation and/or elastase release. 8-Formylalloxanthoxyletin (**1**), alloxanthoxyletin (**4**), and xanthoxyletin (**9**) were the most effective among the isolated compounds, with IC_50_ values of 4.60 ± 0.83/2.59 ± 0.52, 1.47 ± 0.53/3.43 ± 0.63, and 1.47 ± 0.41/4.18 ± 0.73 µg/mL, respectively, against fMLP-induced O_2_^•−^ generation and elastase release. Our study suggests *Z. avicennae* and its isolates (especially **1**, **4**, and **9**) are worthy of further biomedical investigation and could be expectantly developed as potential candidates for the treatment or prevention of various inflammatory diseases.

## Supplementary Materials

Supplementary materials can be found at http://www.mdpi.com/1422-0067/16/05/9719/s1.
