# Supplementary material for: New Coumarin Derivatives and Other Constituents from the Stem Bark of Zanthoxylum avicennae: Effects on Neutrophil Pro-Inflammatory Responses
Source: Int J Mol Sci. 2015 Apr 29;16(5):9719–31. doi: 10.3390/ijms16059719 (PMC4463613; doi:10.3390/ijms16059719)
Supplement: Supplementary file 1 [file ijms-16-09719-s001.pdf]

Supplementary Information

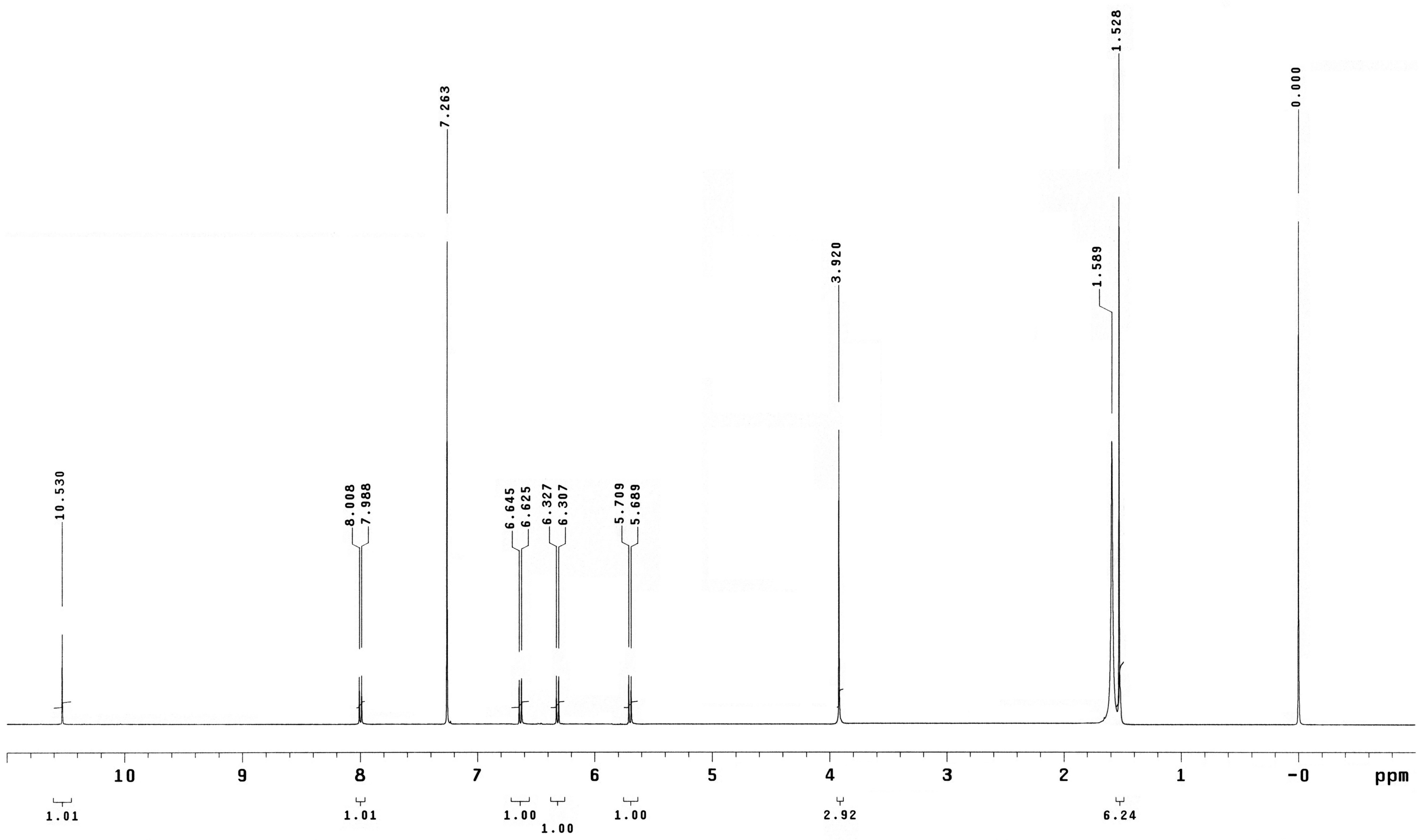

Figure S1. ^{1}H NMR spectrum ($CDCl_3$, 500 MHz) of **1**.

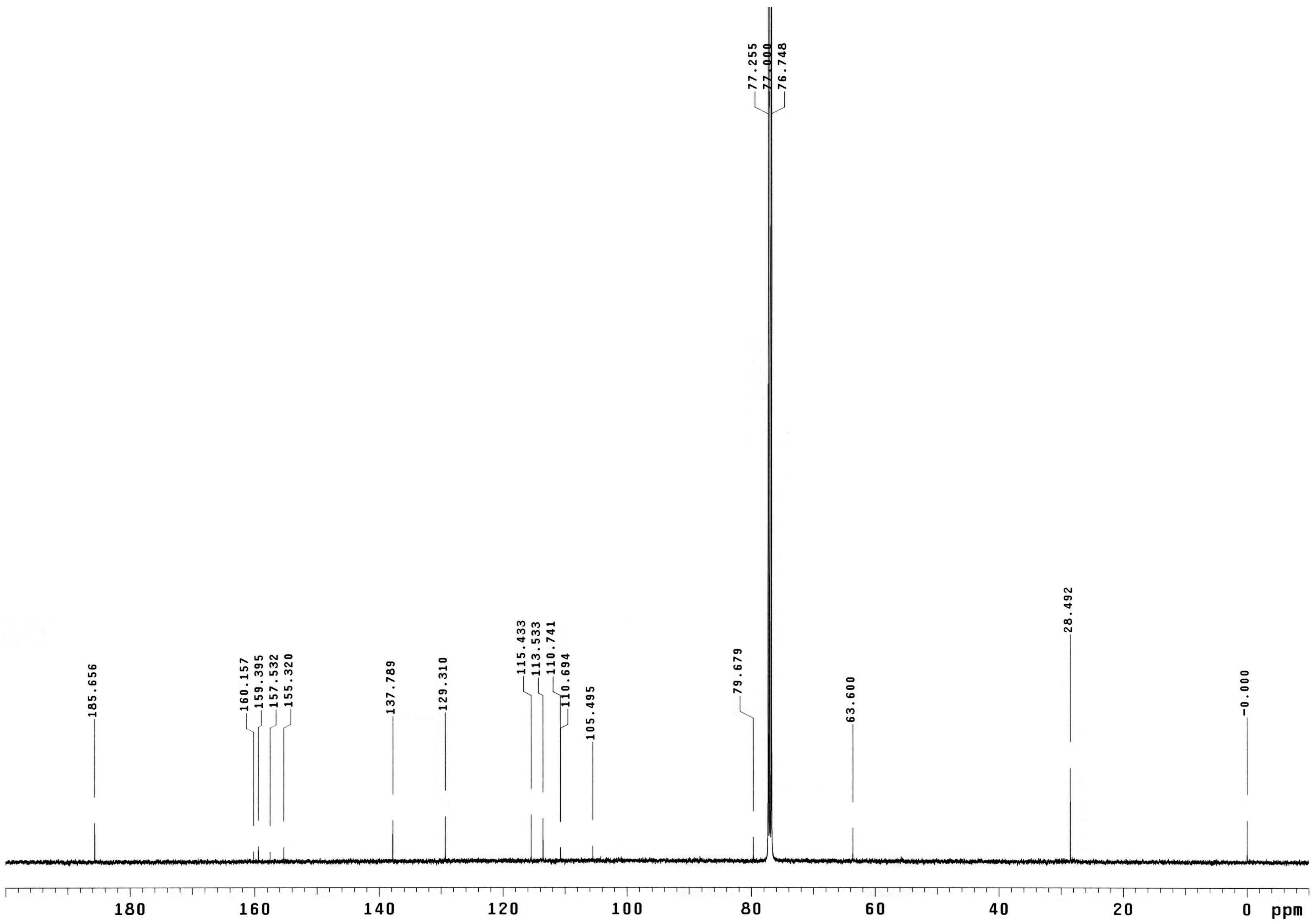

Figure S2. ^{13}C NMR spectrum ($CDCl_3$, 125 MHz) of **1**.

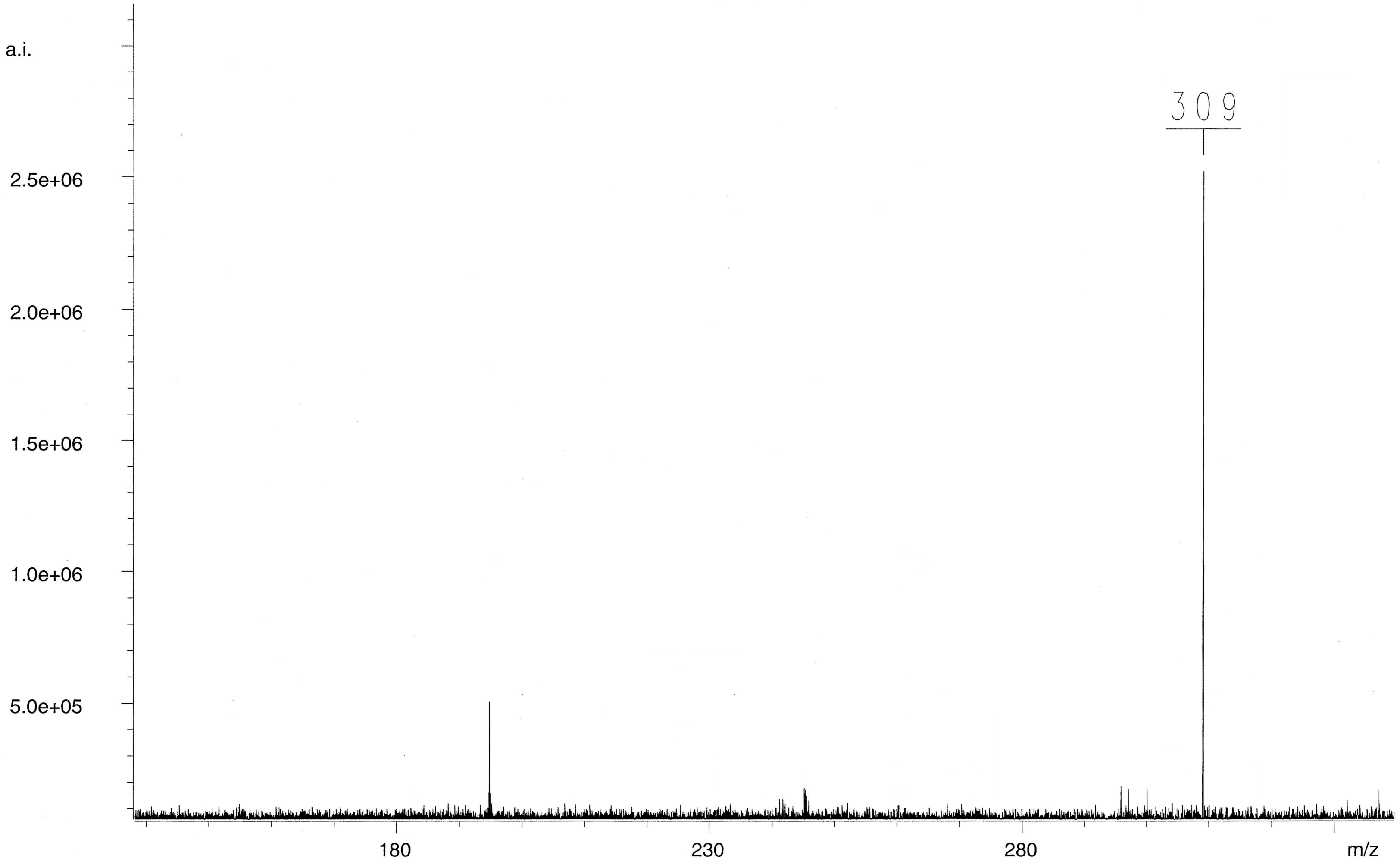

Figure S3. ESI-MS spectrum of **1**.

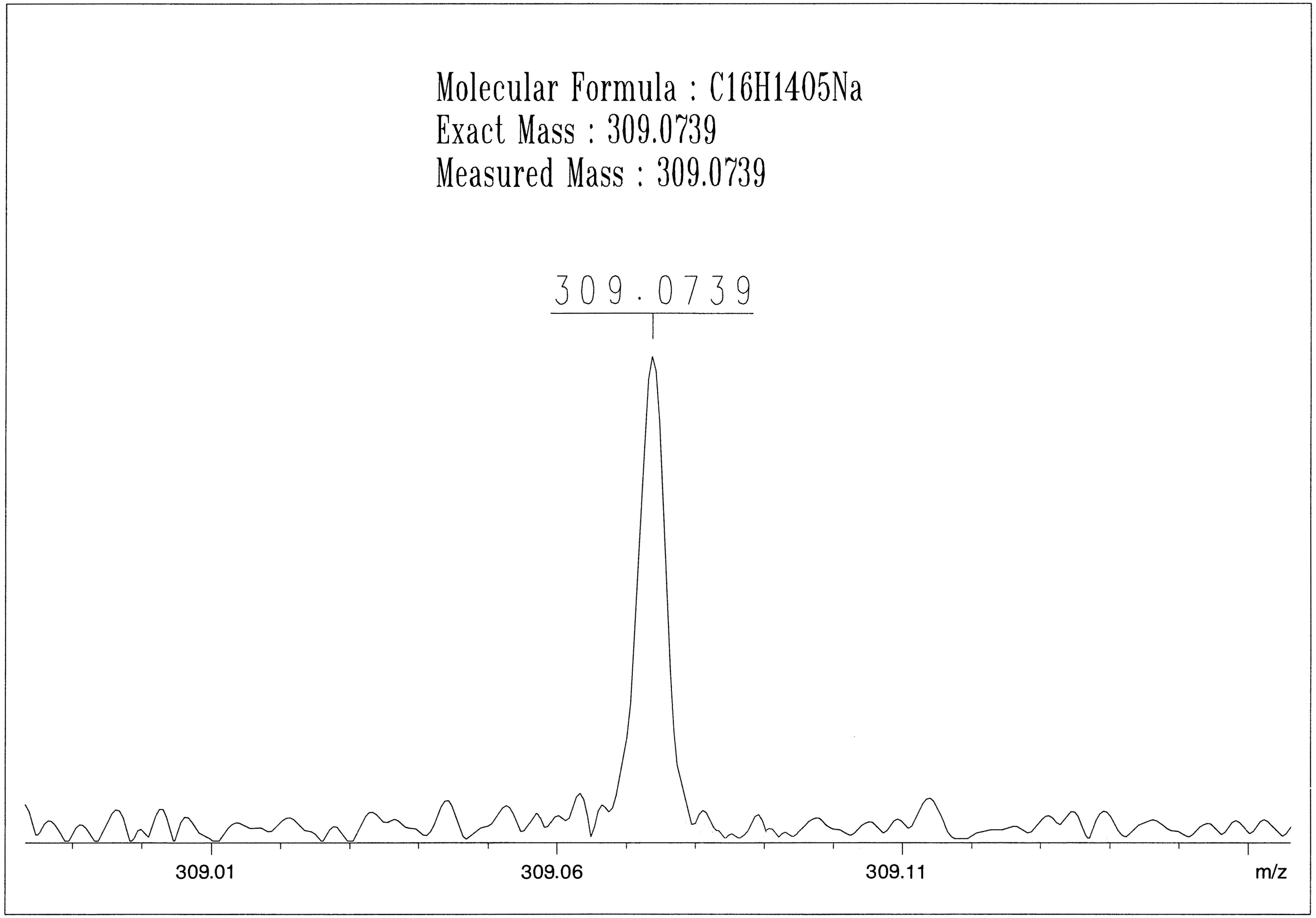

Figure S4. HR-ESI-MS spectrum of **1**.

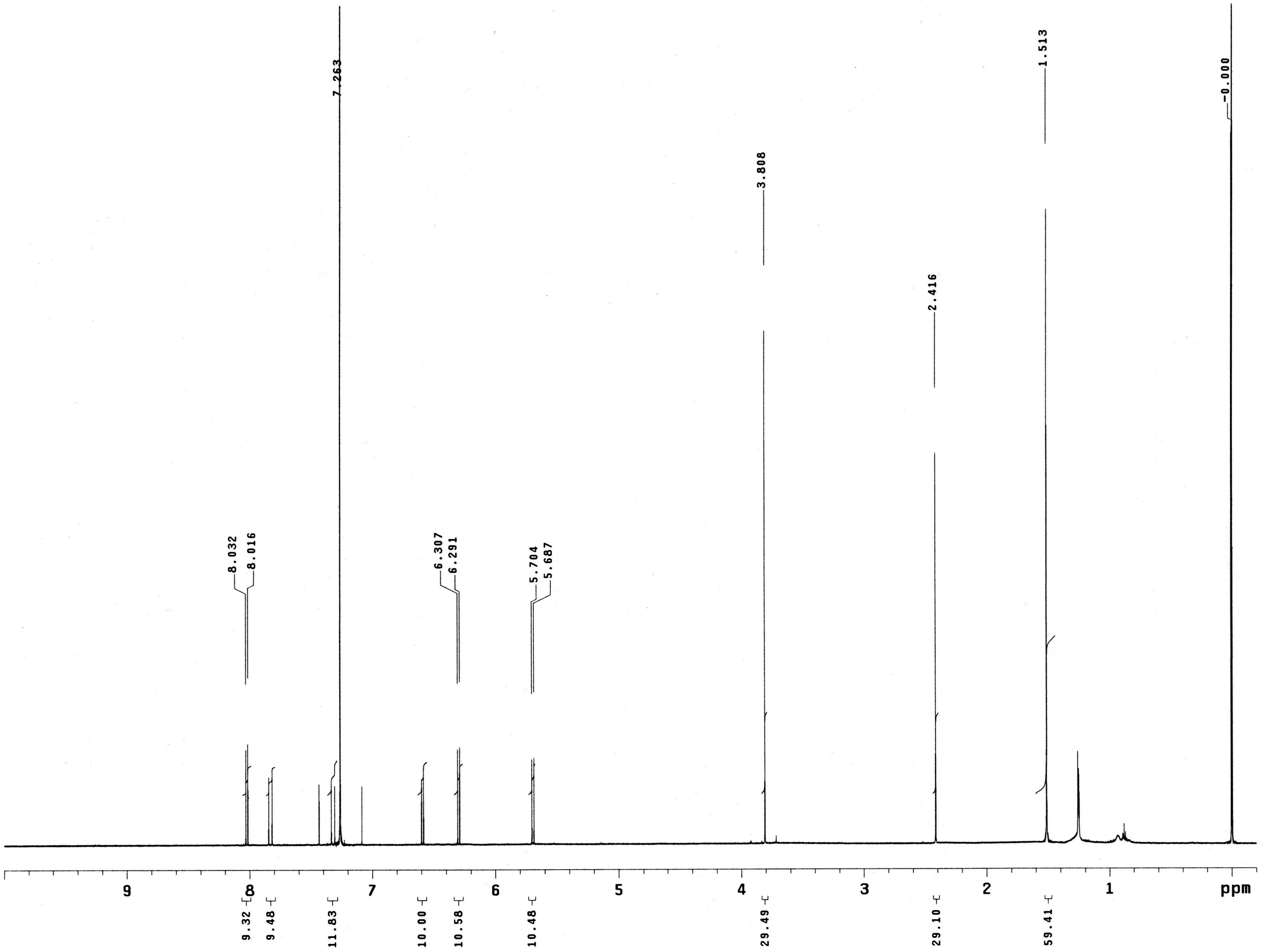

Figure S5. ^{1}H NMR spectrum ($CDCl_3$, 600 MHz) of **2**.

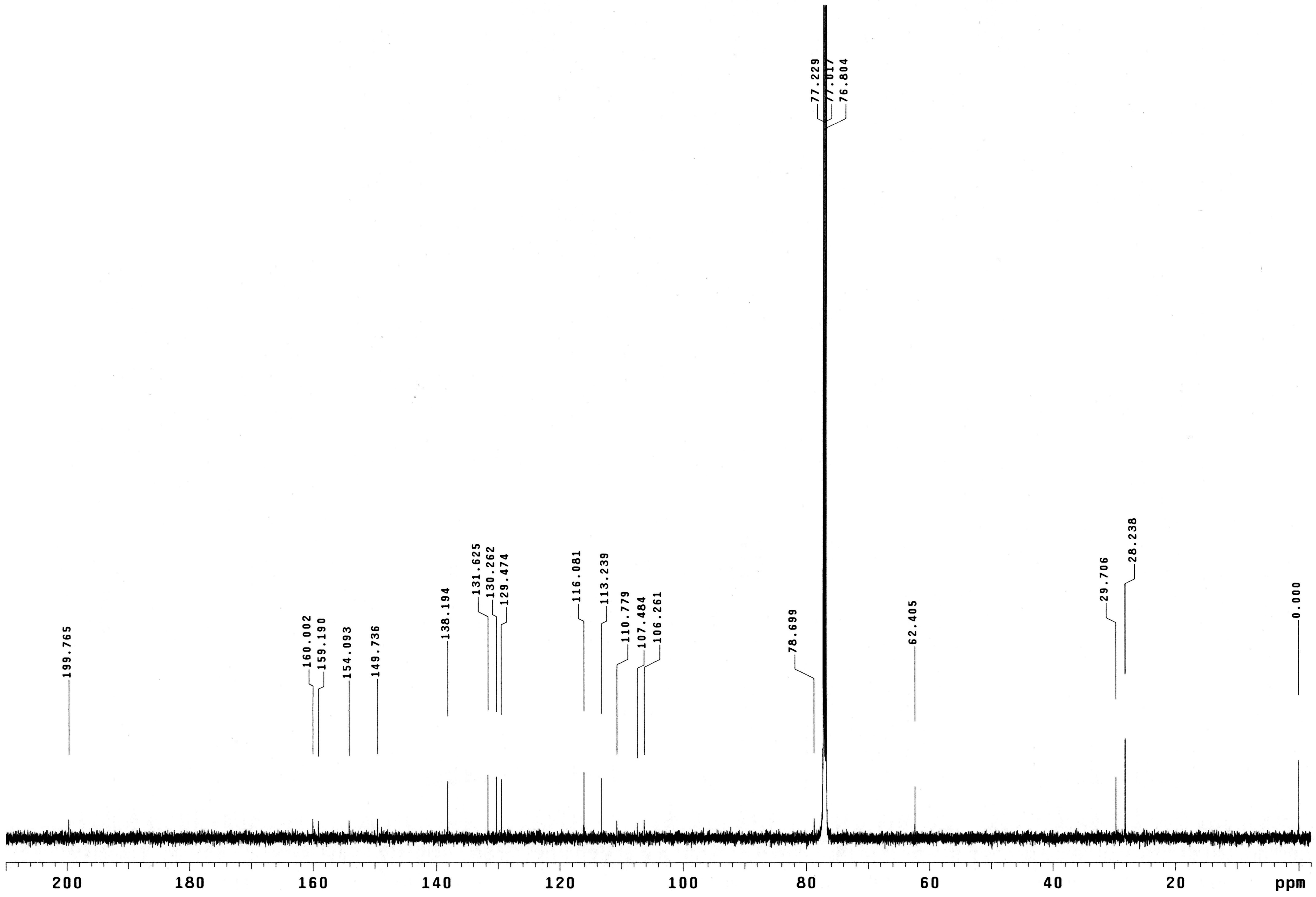

Figure S6. ^{13}C NMR spectrum ($CDCl_3$, 150 MHz) of **2**.

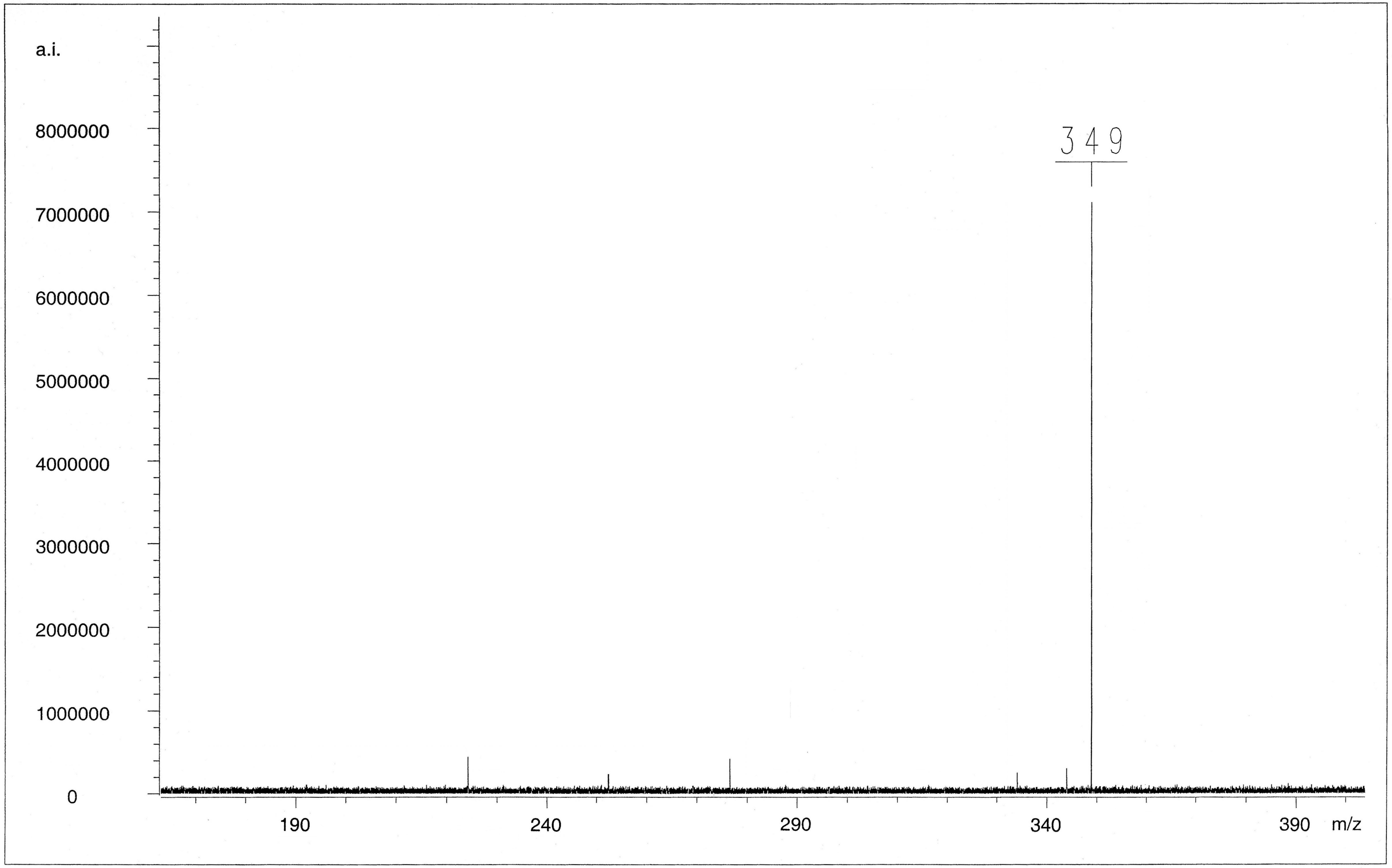

Figure S7. ESI-MS spectrum of **2**.

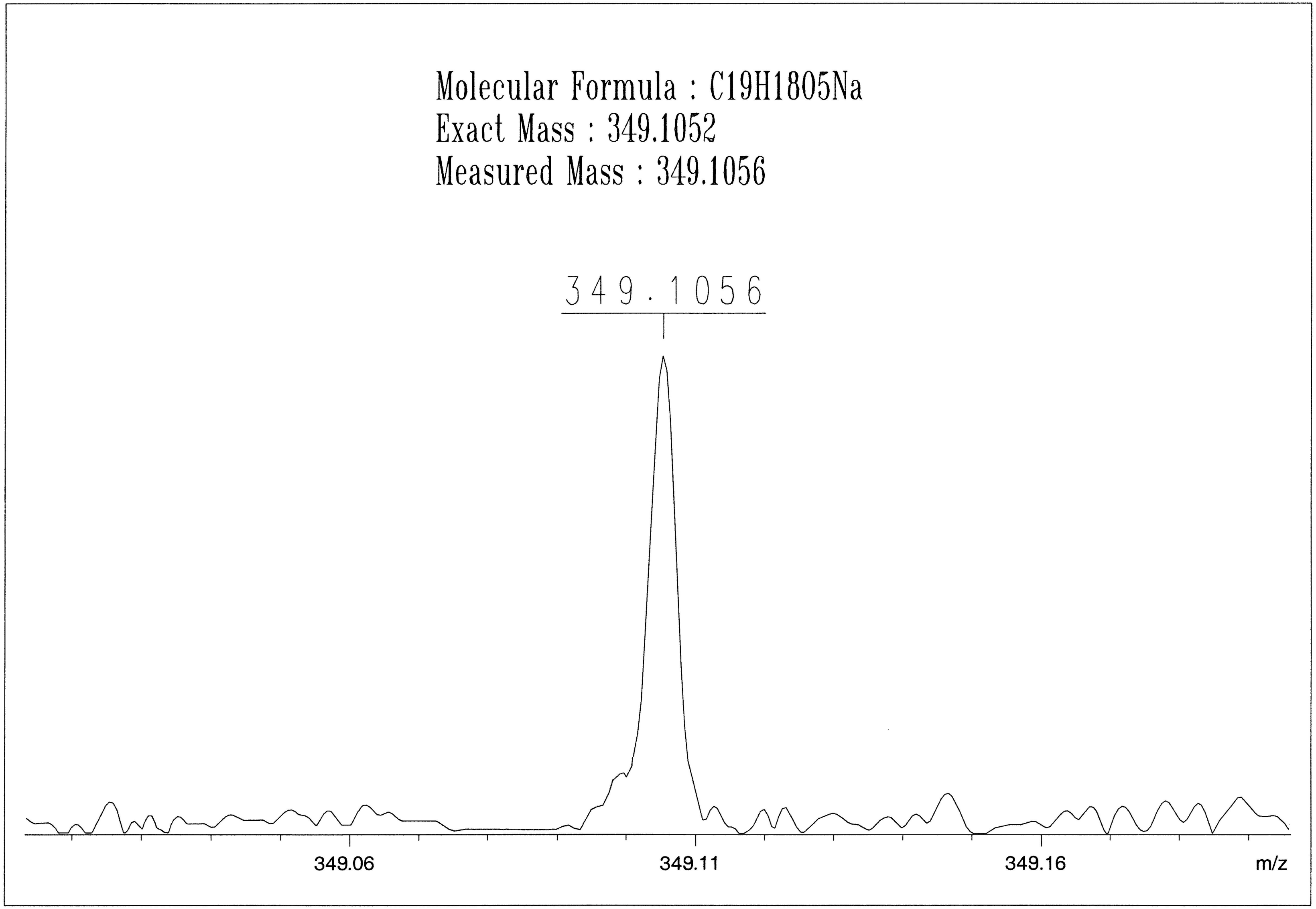

Figure S8. HR-ESI-MS spectrum of **2**.

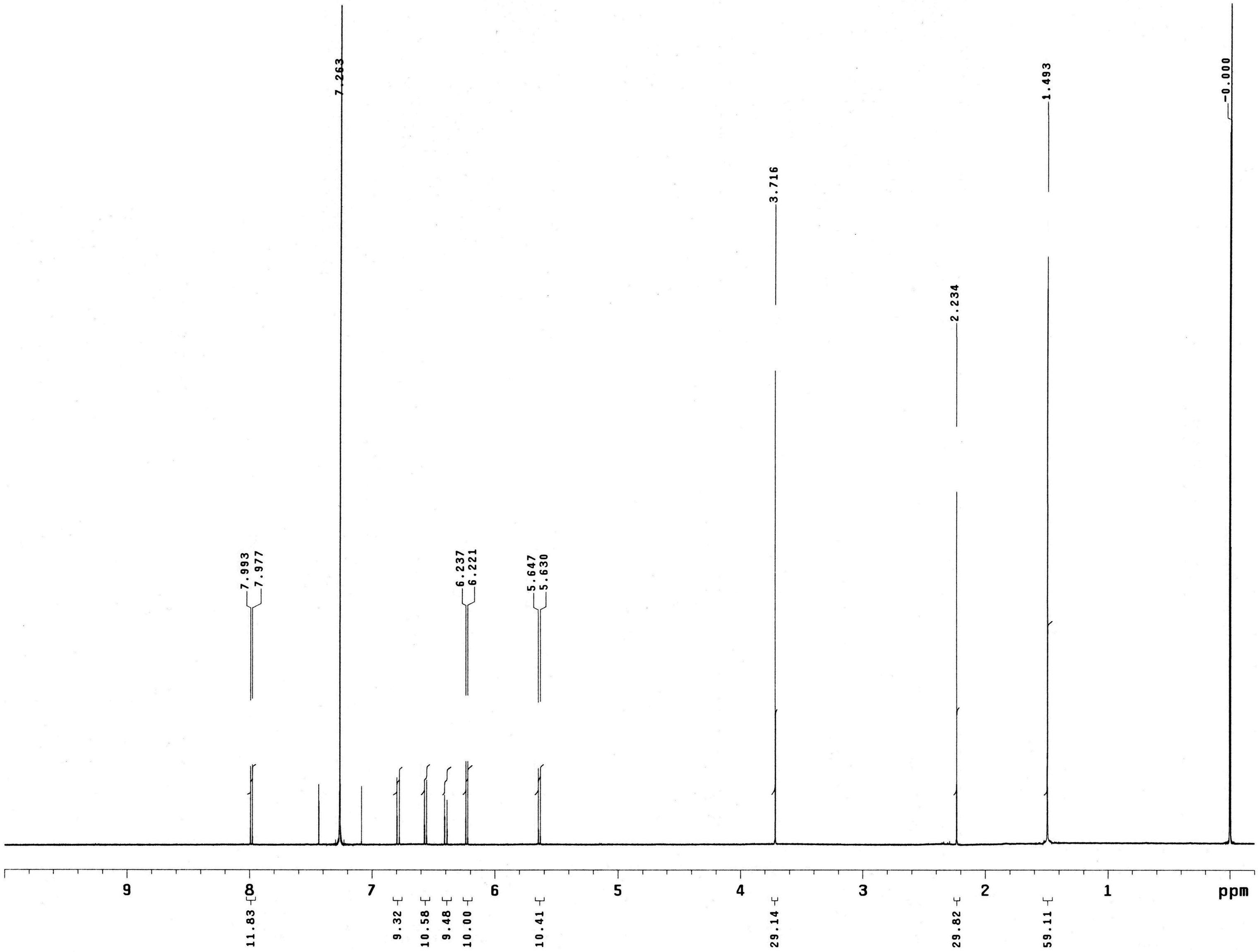

Figure S9. ^{1}H NMR spectrum ($CDCl_3$, 600 MHz) of **3**.

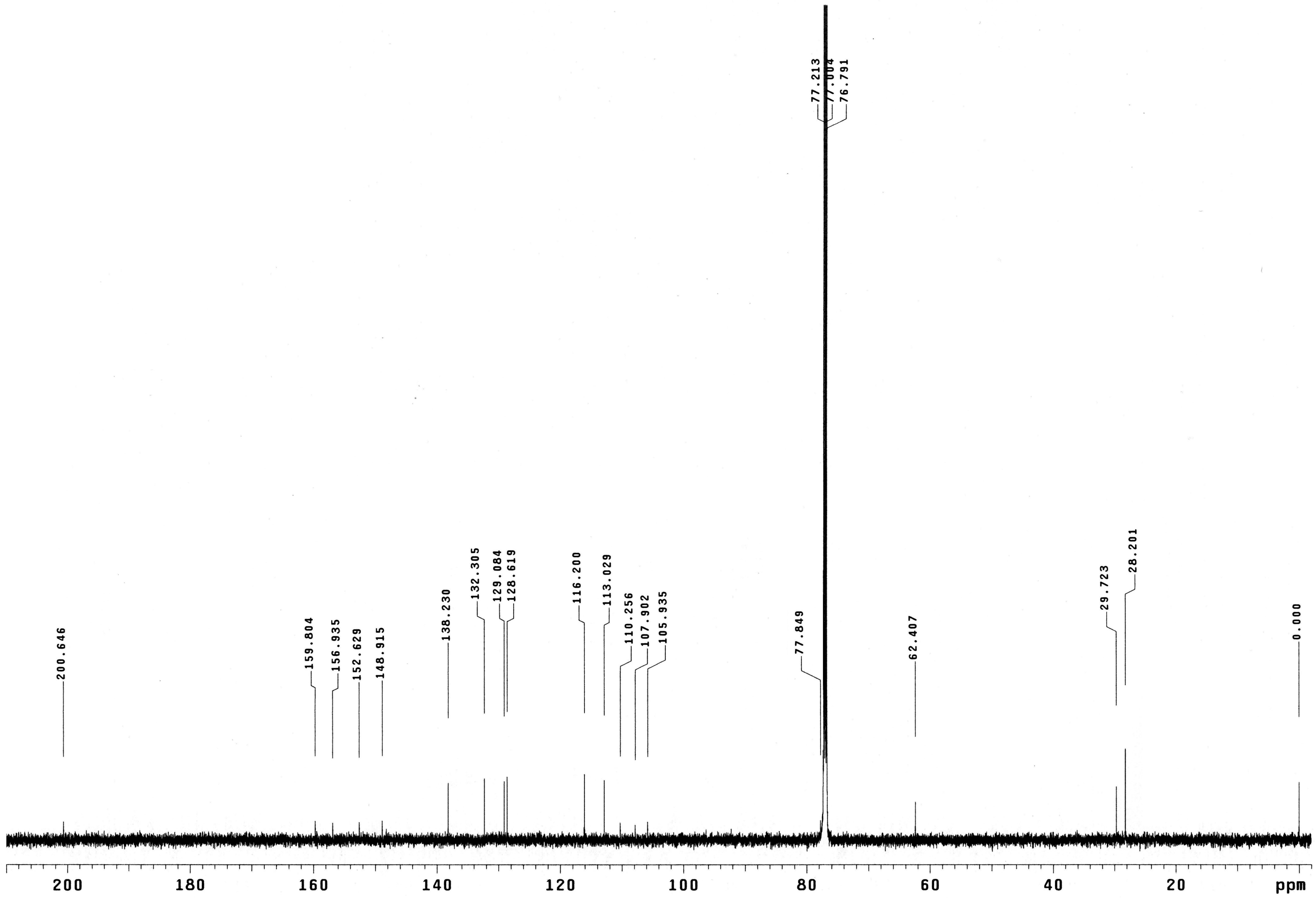

Figure S10. ^{13}C NMR spectrum of **3** ($CDCl_3$, 150 MHz).

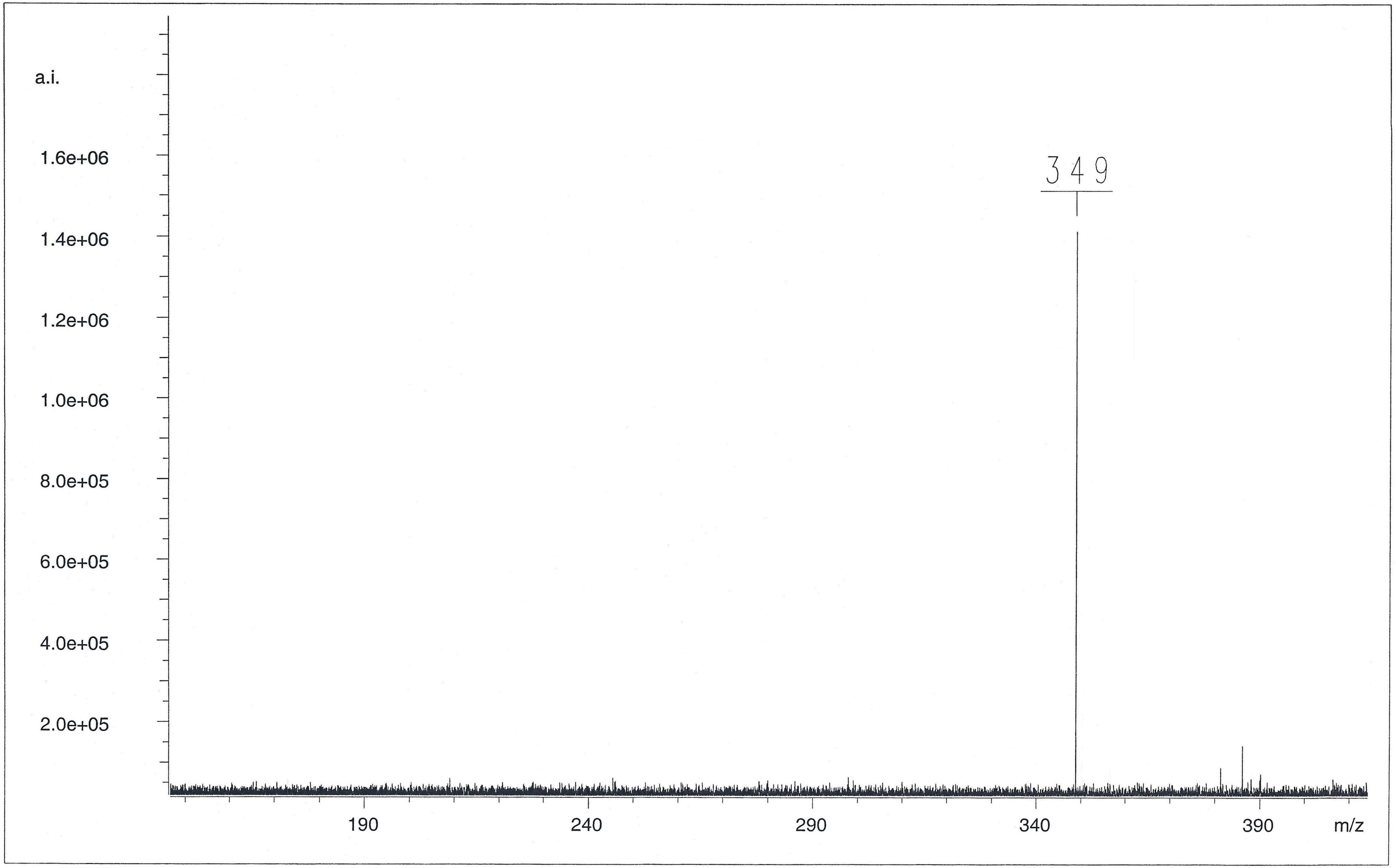

Figure S11. ESI-MS spectrum of **3**.

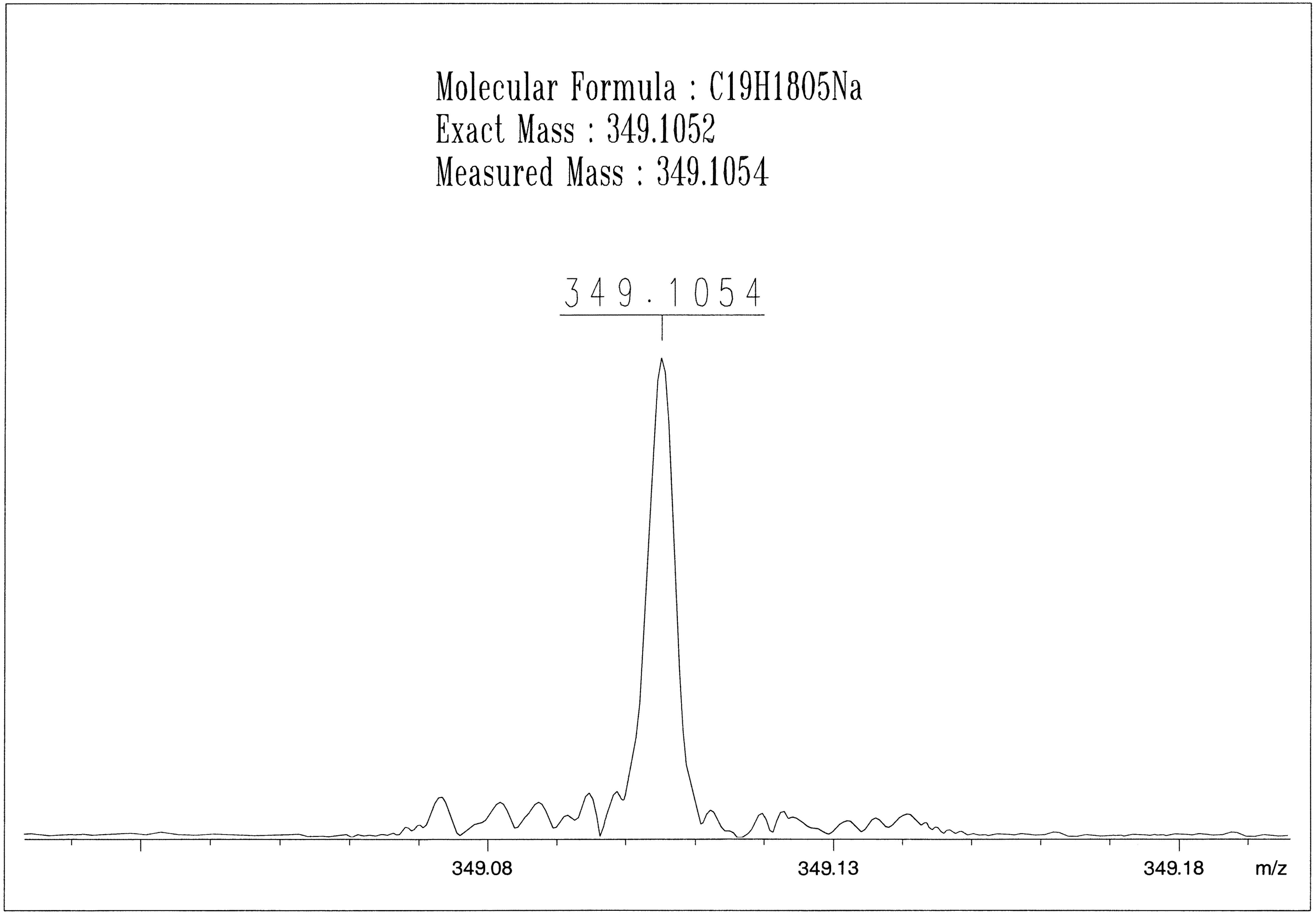

Figure S12. HR-ESI-MS spectrum of **3**.
